# The effect of chronic microplastic exposure on the growth, biochemical responses, and histological changes of the juvenile sea cucumber *Holothuria scabra*

**DOI:** 10.1007/s11356-025-36559-1

**Published:** 2025-06-04

**Authors:** Sarah Syazwani Shukhairi, Nurzafirah Mazlan, Nur Nashrah Abd Rahman, Muhammad Nor Afdall Nazahuddin, Amir Syazwan Shawel, Vijay Subbiah Kumar

**Affiliations:** 1 Higher Institution Centres of Excellence (HICoE), Borneo Marine Research Institute, Universiti Malaysia Sabah, Jalan UMS, 88400 Kota Kinabalu, Sabah Malaysia; 2 Biotechnology Research Institute, Universiti Malaysia Sabah, Jalan UMS, 88400 Kota Kinabalu, Sabah Malaysia

**Keywords:** Echinoderms, Microplastics, *Holothuria scabra*, PMMA, Chronic toxicity

## Abstract

Microplastics (MPs) are minuscule plastic particles less than 5 mm in size, originating from the degradation of larger plastic debris. MPs originate from various sources and pose a significant threat to the marine ecosystem. *Holothuria scabra* is a species of sea cucumber with high commercial value and among the organisms affected by MPs pollution. *H. scabra* are also crucial in maintaining a clean seabed and recycling nutrients in the ocean ecosystem. Polymethymethacrylate (PMMA), a durable and transparent plastic polymer widely used as a glass alternative in maritime and other industries. This research aimed to investigate the toxicity effects of polymethylmethacrylate-MPs on the well-being of juvenile sea cucumber *H. scabra*. Over a 60-day treatment period, polymethylmethacrylate MPs were exposed to the juvenile sea cucumber diet at concentrations of 0.6 MPs/g (Treatment 1), 1.2 MPs/g (Treatment 2), and 10 MPs/g (Treatment 3) while a control group which received no MPs exposure to observe changes in their growth, biochemical responses, and histological alteration. The mean weight, weight gain percentage and specific growth rate exhibited significant differences (*p* < 0.05) with the control group displaying the highest SGR value of 1.22 ± 0.35%. Mortality was observed in treatment 2 and 3, respectively. A disruption in enzyme activity was also observed across all treatment groups (*p* < 0.05). The findings of growth rates and biochemical responses were further supported by histological observation, uncovering injuries and loss of cellular components in respiratory trees and intestines. This study enhances our understanding of the toxicity mechanism associated with PMMA-MPs in deposit feeder organisms.

## Introduction

Microplastics (MPs), ranging in size less than 5 mm, have become prevalent in oceans globally, spanning from surface to sediments. MPs are either produced directly during manufacturing or secondarily through the degradation of larger plastic materials (Frias and Nash 2019). Primary MPs sources are particles that are manufactured at small sizes for commercial applications such as microbeads from cosmetics, personal care goods, and nurdles. As for secondary MPs, it is formed when bigger plastics undergo degradation and fragmentation in the environment, including mechanical factors and chemical factors over a period of time. The MPs abundance has been discovered in surface water and sediments in Malaysia (Khalik et al. 2018; Noik et al. 2015; Sarijan et al. 2018). There is a growing realization that MPs are contaminating seafood as a result of their uptake in habitats, processing, or packaging (Akhbarizadeh et al. 2020; Cox et al. 2019; Mohsen et al. 2023; Webb et al. 2019). These contaminants will eventually enter the human digestive system as they are being consumed and deposited on the organs. Previous studies on MPs contamination in seafood has been reported in fish (Giani et al. 2019; Lopes et al. 2020), shrimp (Gurjar et al. 2021; Hossain et al. 2020), sea cucumber (Muhammad Husin et al. 2021; Mazlan et al. 2023b), bivalves (Abd Rahman et al. 2024; Li et al. 2022) and zooplankton (Ngo et al. 2023). However, no reports on the adverse effects of the MPs towards growth and ecosystem of the marine animals.

PMMA has been used extensively in diverse industries ranging from automotive and biomedical applications due to its transparency, rigidity and resistance to environmental degradation. Its durability also makes it a persistent pollutant in the marine environment, where it is favored in the maritime sector as a glass substitute due to its high impact strength and shatter resistance (Mazlan et al. 2023b). The selection of PMMA MPs in this research was critical as they exemplify durable, non-biodegradable polymers deposited in marine animals’ guts. However, the toxicological effects of PMMA on these animals remain largely unexplored.

Holothurians are marine invertebrates that are members belonging to Phylum Echinodermata and are also referred to as sea cucumbers. These deposit feeders are often found on the seabed, utilizing their ability to convert the habitat’s substrate into a food source (Manuputty et al. 2019). Many sea cucumbers are commercially harvested and dried for human consumption or medical purposes, particularly in Asian nations (Mazlan et al. 2023a). Sea cucumber offers an exceptional nutritional profile that includes vitamins, minerals, and amino acids as well as pharmacological actions which include wound healing, anti-inflammatory, and antioxidant (Bordbar et al. 2011; Shi et al. 2016).

*Holothuria scabra* is one of the rare tropical species that prefers ordinary coastal areas to coral reefs and muddy sand habitats. Due to their daily burrowing cycle and non-selective feeding behaviours, sea cucumbers generally move sluggishly and often bury themselves in the sediment. The increasing demand of *H. scabra* products in Sabah primarily involves dried sea cucumbers which are highly valued in international markets, particularly in East and Southeast Asia. The growing demand for these products has led to overexploitation of wild populations with the species now classified as “Endangered” by the International Union for Conservation of Nature (IUCN-2010) and highlighting the need for sustainable aquaculture practices, particularly in Sabah. As the increased demand and value of sea cucumber products pose a significant threat to wild populations, sea cucumber mariculture offers a pathway for the restoration of wild stocks (Purcell et al. 2014). However, these aquaculture environments cause the widespread issue of MPs, which are prevalent in coastal areas and sediment. These sea cucumbers inadvertently consume MPs as they use their tentacles to collect sediments, which eventually leads to an increase in the intake of plastics. In their natural habitat, sea cucumbers fulfil their nutrient requirements by consuming and digesting algae and organic debris in sediments, including those contaminated with microplastics (Purcell et al. 2014). This ecological dynamic also affects sea cucumbers in pond cultivation, given that the sediments used are sourced from the natural environment, an inevitable factor contributing to potential exposure to MPs. Research findings indicate various adverse impacts of MPs on sea cucumbers.

Studies show that MPs can cause oxidative stress, neurotoxicity and immunotoxicity in marine animals. Zhong et al. (2024) revealed that bio-based and traditional petroleum-based polystyrene MPs cause similar harmful effects on mussels, leading to physiological stress and disturbance in enzyme activities. The study demonstrates that polypropylene MPs and chemical pollutants work together to cause inflammation in the intestines and gut microbiota imbalance in European sea bass *Dicentrarchus labrax* (Montero et al. 2022). Han et al. (2021) revealed that polystyrene MPs and seawater temperature synergistically impair survival, growth, gut structure and immune function in brine shrimp *Artemia franciscana*. In marine invertebrates like sea cucumbers, which lack adaptive immunity, the innate immune system plays a vital role in defense against pathogens. Enzymatic activities, particularly those related to oxidative stress and immune defense, are key components of this innate response. Studies have shown that enzyme activities are important in the immune defense mechanisms of echinoderms (Chen et al. 2018). These enzymes subsequently respond to environmental stressors in sea cucumbers, such as pH variation and hypoxic stress. Histological analysis also showed abnormalities in the respiratory tree caused by the penetration of MPs (Mohsen et al. 2021b). Furthermore, histological analysis provides insights into the structural integrity of tissues and organs which reveal the severity of MPs toxicity and often complement biochemical findings.

Previous studies have shown that MPs have been detected in sea cucumbers, but their toxicological impact is not fully understood (Iwalaye et al. 2020; Liu et al. 2022; Mohsen et al. 2019; 2021b). MPs intake may negatively affect the physiological functions, biochemical processes and tissue structures of sea cucumbers. This study aims to support the sustainability of sea cucumber *H. scabra* by investigating the toxicity of PMMA-MPs in the sea cucumber. This research focuses on understanding how 60 days of exposure to varying PMMA-MPs concentrations affect the growth rates, enzyme activities and tissue structures of *H. scabra*. The findings will provide valuable insights and deepen understanding into their response and adaptation to PMMA-MPs-induced stress.

## Materials and methods

### Experimental animal

The experiment was performed in the Integrated Multi-Trophic Aquaculture (IMTA) hatchery at Borneo Marine Research Institute, Universiti Malaysia Sabah, Sabah, Malaysia. Sea cucumber *H. scabra* (*n* = 48) were obtained from a hatchery, located in Tuaran with an average weight of 15 to 18 g. The study animals were acclimated in a water recirculation tank supplied with aeration and fed with sargassum powder for one week before the experiment.

### Experimental setup

Polymethylmethacrylate (PMMA-MPs) used for construction sites were obtained from the Crustacean Hatchery of Universiti Malaysia Sabah and was prepared using a grinder and sieved using an 870-µm sieve. The polymers of MPs were identified using Fourier Transform Infrared Spectroscopy (FTIR). The experimental animals were divided into four groups (including control)with three replicates. The stocking density was four animals per m^3^. The animal feed was composed of Sargassum powder at 3% biomass. For control group, the tanks underwent water exchange with filtered water and were fed with sargassum powder without any MPs treatment. *H. scabra* were exposed to the diet that was mixed with MPs in three treatments of 0.6 MPs/g (Treatment 1), 1.2 MPs/g (Treatment 2) and 10 MPs/g (Treatment 3) for 60 days to investigate the effects of chronic PMMA-MPs exposure on the growth and physiological status of juvenile sea cucumber (Mohsen et al. 2021b). In Treatment 1 and Treatment 2 groups, the diet was mixed with MPs at concentrations reflecting environmental levels, while in treatment 3 included MPs at higher concentrations simulating a worst-case scenario, reflecting the increasing accumulation of MPs in the ocean (Mohsen et al. 2019, 2021a). The amount of MPs added to the feed for each treatment group was precisely measured to ensure the correct and consistent dosage. Additionally, feeding time for all groups was standardized. Sixty days of chronic exposure is considered adequate to evaluate the toxicity of sea cucumber among different treatment groups (Bai et al. 2018; Mohsen et al. 2021b).

### Rearing conditions

The juvenile sea cucumbers were distributed in tanks provided with aeration. The water temperature was 29 to 30 °C, which was the optimum temperatures for the growth of sea cucumbers. The pH was 7 to 8 and the salinity was 33 to 34 ppt. Four juvenile sea cucumbers were stocked per tank in a 1 m^3^ tank. During the experiment, sea cucumbers were fed once a day and the 50% water exchange was done daily.

### Growth rate evaluation

The impact of dietary exposure to PMMA-MPs on the growth rate of juvenile sea cucumber was evaluated according to Bai et al. (2018) and Mohsen et al. (2021b). Before the experiment, juvenile sea cucumbers of similar size were selected and weighed after allowing them to rest for two minutes to expel excess water and gently patted dry with a soft cloth to remove any remaining moisture before being distributed across the tanks. After two days after their last meal following 60 days of treatment, sea cucumbers were weighed. Weight gain percentage and specific growth rate (SGR) were calculated according to the following equations:

$$\begin{array}{l}\mathrm{Weight}\;\mathrm{Gain}\;\mathrm{Percentage}(\%)=\left(\mathrm{Final}\;\mathrm{Weight}-\mathrm{Initial}\;\;\mathrm{Weight}\right)\times100\\\mathrm{Specific}\;\mathrm{Growth}\;\mathrm{Rate}\left(\mathrm{SGR}\right)=100\times\frac{\left(InFW-InIW\right)}T\end{array}$$

where IW and FW were the initial and final body weights of sea cucumbers, and T was the duration of the experiment (Bai et al. 2018; Mohsen et al. 2021b).

### Enzyme assay analysis

The enzymes activities were studied to examine the physiological condition of *H. scabra* after prolonged PMMA-MPs exposure. Coelomic fluid was collected in test tubes and stored at − 80 °C until further analysis (Mohsen et al. 2021b). The immune enzyme acid phosphotase, digestive enzyme lipase, and oxidative enzymes malondialdehyde and superoxide dismutas were evaluated spectrophotometrically using commercial kits.

Acid phosphatase (ACP) activities were determined spectrophotometrically at a wavelength of 405 nm with the Acid Phosphatase Activity assay kit (p-nitrobenzene phosphate (PNPP) method) obtained from ElabScience (USA). Acid phosphatase converted disodium p-nitrobenzene phosphate, a chromogenic substrate for phosphatase into producing p-nitrophenol. P-nitrophenol exhibited a yellow color in alkaline environments, with a peak absorption at 405 nm.

Digestive enzyme lipase was measured at 710 nm using commercial kits (Macklin (China)). Lipase catalyzed the hydrolysis of triglycerides into fatty acids and glycerol, producing a blue color. The activity of lipase was expressed as μmol/min/mL and the amount of oil hydrolyzed per millimeter per minute at 37 °C provide 1 μmol fatty acid.

Malondialdehyde (MDA) and superoxide dismutase (SOD) were measured at 532 nm and 600 nm, respectively using kit produced from Macklin (China). Oxygen free radicals interacted with unsaturated fatty acids in lipids, resulting in the production of peroxidized lipids. This process gradually transformed into a complex series of compounds, including MDA. MDA reacted with thiobarbituric acid to generate a red product with a maximum absorption peak of 532 nm. Through colorimetry, the peroxidized lipid content in the sample was estimated. Simultaneously, the absorbance at 600 nm was measured. The MDA content was then calculated by determining the difference in absorbance between 532 and 600 nm. The activity of MDA was expressed as nmol/mL.

SOD was measured using commercial kits (Macklin (China)). SOD catalyzed disproportionation of superoxide anion to form hydrogen peroxide (H_2_O_2_) and oxygen (O_2_). Superoxide anion (O_2_-) was produced by the xanthine and xanthine oxidase reaction system. O_2_- ions reduced blue tetrazole to form blue formazan, which has an absorbance of 560 nm. SOD removed O_2_- and inhibit the formation of methionine. Samples were diluted tenfold before the determination procedure. SOD activity was expressed U/mL represents the amount of enzyme catalyzing 50% inhibition in the reaction system of the xanthine oxidase.

### Histological analysis

The respiratory trees and the intestines of the juvenile sea cucumber *H. scabra* were removed carefully from the animal and fixed into a 10% neutral buffered formalin solution. All the tissues underwent tissue processing and were embedded in paraffin blocks. Then, the samples were sectioned into 5-μm thickness and stained with Haematoxylin and Eosin (H & E) staining (Lei et al. 2018). The slides were examined under a microscope with an attached camera (Mohsen et al. 2021b).

### Statistical analysis

One-way ANOVA followed by Tukey’s test for multiple comparisons was used to compare the specific growth rate and enzyme activities between four experimental treatments. Differences were accepted as significant if *p* < 0.05. Values are presented as mean ± standard error (SE). All statistical analyses were performed with software SPSS version 29.

## Results

### Growth rate evaluation

**Fig. 1:**
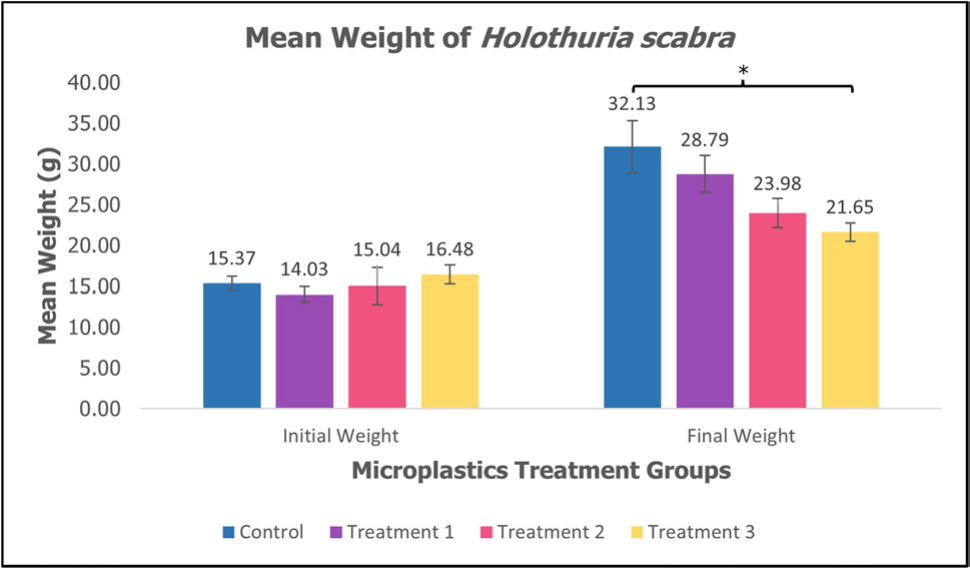
Mean weight (mean ± SE) of sea cucumber *H. scabra*. *Significant difference (*p* < 0.05, *p* = 0.01)

**Fig. 2:**
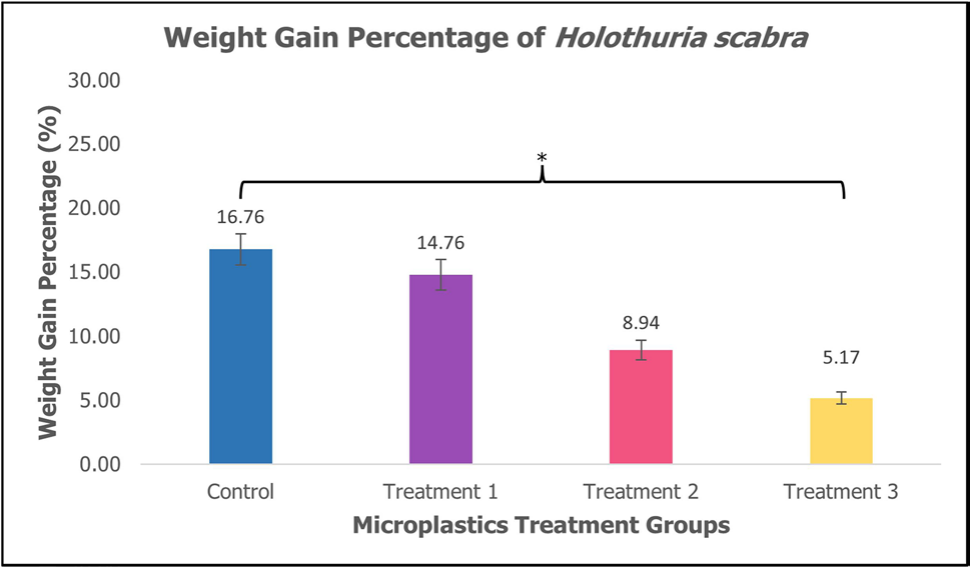
Weight gain percentage (mean ± SE) of sea cucumber H. scabra. *Significant difference (*p* < 0.05, *p* = 0.032)

**Fig. 3:**
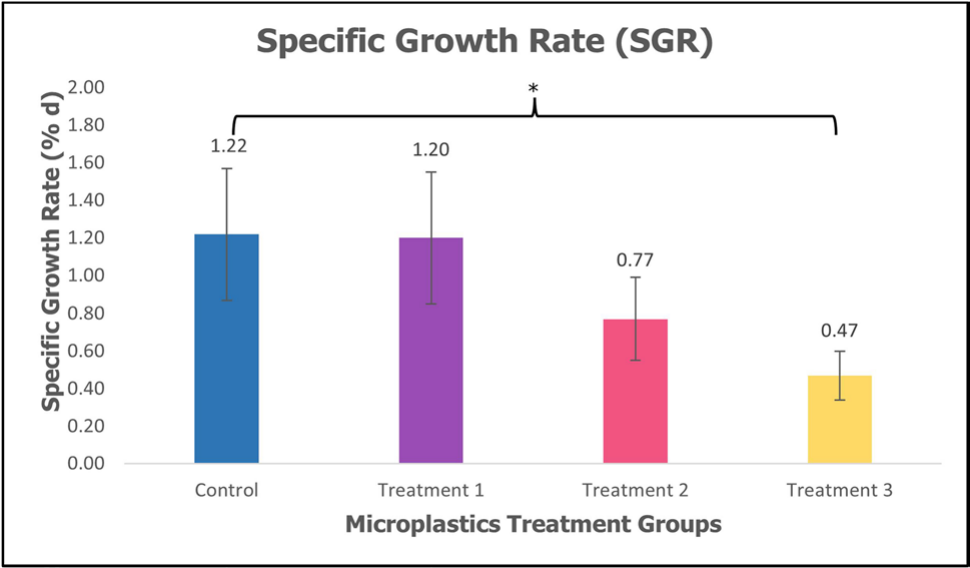
Specific growth rate (SGR) (mean ± SE) of sea cucumber *H. scabra*. *Significant difference (*p* < 0.05, *p* = 0.03)

Juvenile sea cucumber *H. scabra* were fed with MPs in their diet for 60 days. The mean weight, weight gain percentage, and specific growth rate differed significantly in the control group compared to the treatment groups as shown in Table 1. The mean weight (mean ± SE) of sea cucumber *H. scabra* decreases as the concentration of MPs increases with a significant difference (*p* < 0.05, *p* = 0.01) as shown in Fig. 1. The weight gain percentage (mean ± SE) of sea cucumber *H. scabra* showed a decrease between treatment groups compared to control groups with a significant difference (*p* < 0.05, *p* = 0.032) as shown in Fig. 2. Figure 3 depicts specific growth rate (SGR) (mean ± SE) of sea cucumber *H. scabra* which decreases significantly (*p* < 0.05, *p* = 0.03) as the MP concentration increases. The survival rate was 100% for the control and Treatment 1 group, however, 75% for Treatment 2 and 3 with 3 animals lost, respectively. Unfortunately, there were several sea cucumbers from Treatment 2 and Treatment 3 showed signs of skin lesions disease before 2 days of starvation period and evisceration was also observed.

**Table 1:** The initial body weight, final body weight, weight gain percentage and specific growth rate of *Holothuria scabra* (mean ± SE)

Treatments	Initial W (g)	Final W (g)	Weight gain percentage (%)	SGR (% d)
Control	15.37 ± 0.87	32.13 ± 3.20	16.76 ± 0.35	1.22 ± 0.35
Treatment 1	14.03 ± 0.94	28.79 ± 2.24	14.76 ± 0.35	1.20 ± 0.35
Treatment 2	15.04 ± 2.27	23.98 ± 1.78	8.94 ± 0.22	0.77 ± 0.22
Treatment 3	16.48 ± 1.16	21.65 ± 1.13*	5.17 ± 0.13*	0.47 ± 0.13*

*Different superscript letters within the column indicated significant value of (*p* < 0.05)

### Enzyme assay analysis

**Fig. 4:**
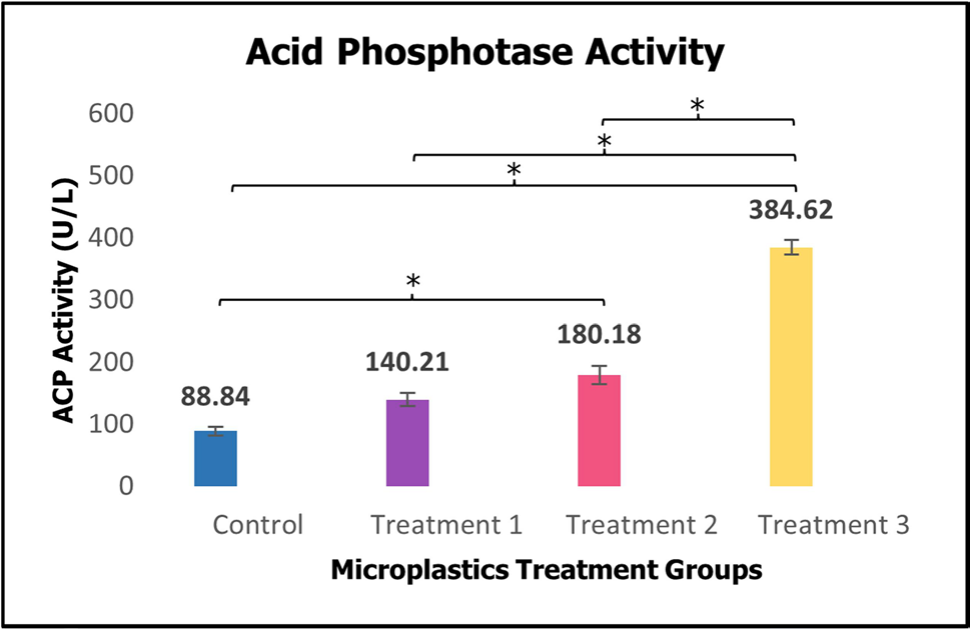
Activity of immune enzyme acid phosphatase (ACP) of sea cucumber *Holothuria scabra* after 60 days of microplastics treatments (mean ± SE). *Significant difference (*p* < 0.05, *p* = < 0.001)

Acid phosphatase (ACP) levels in juvenile *H. scabra* were significantly higher in Treatment 3 (*p* < 0.05, *p* = < 0.001) compared to the control group over 60 days of the treatment period (Fig. 4).

**Fig. 5:**
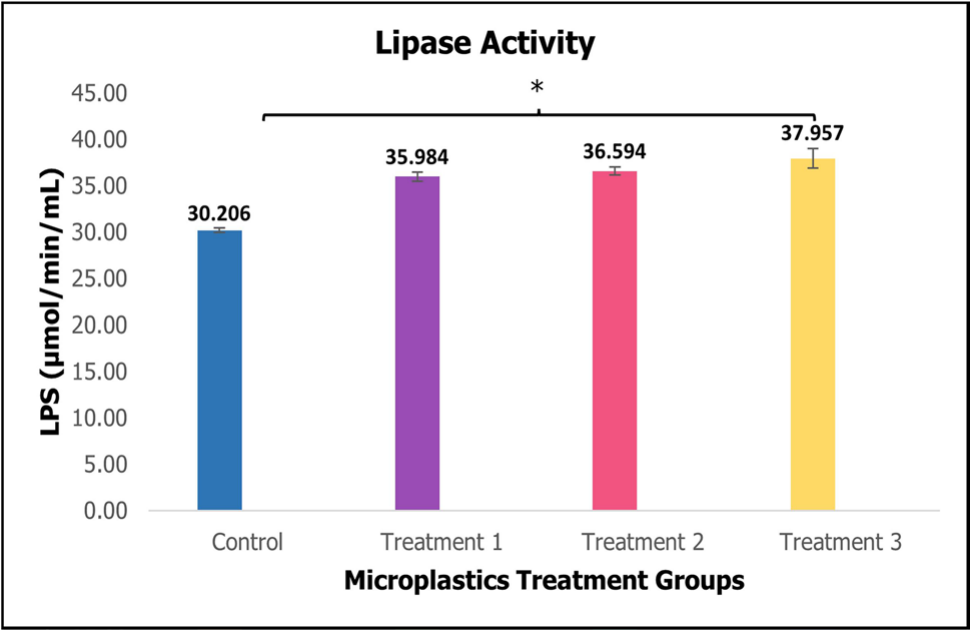
Activity of digestive enzyme lipase of sea cucumber *Holothuria scabra* after 60 days of microplastics treatments (mean ± SE). *Significant difference (*p* < 0.05, *p* = 0.022)

Lipase levels in these juvenile *H. scabra* exhibited a slight increase in the treatment groups compared to the control groups, as depicted in Fig. 5 with a significant difference.

**Fig. 6:**
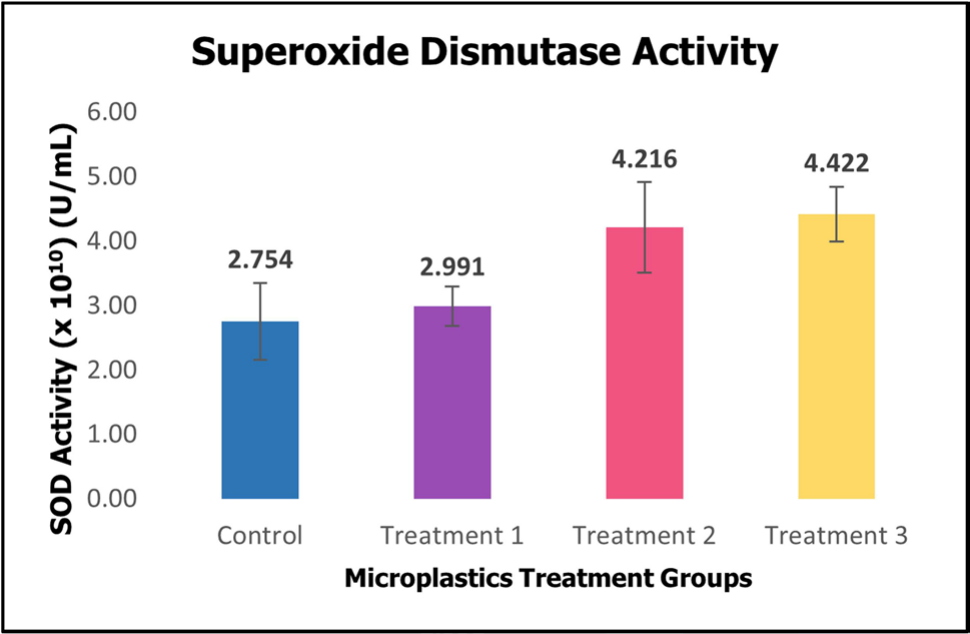
Activity of oxidative enzyme superoxide dismutase (SOD) of sea cucumber *Holothuria scabra* after 60 days of microplastics treatments (mean ± SE). *Significant difference (*p* < 0.05, *p* = 0.047)

Superoxide dismutase (SOD) activity in Fig. 6 demonstrated an increase in the treatment groups. However, the difference did not reach statistical significance.

**Fig. 7:**
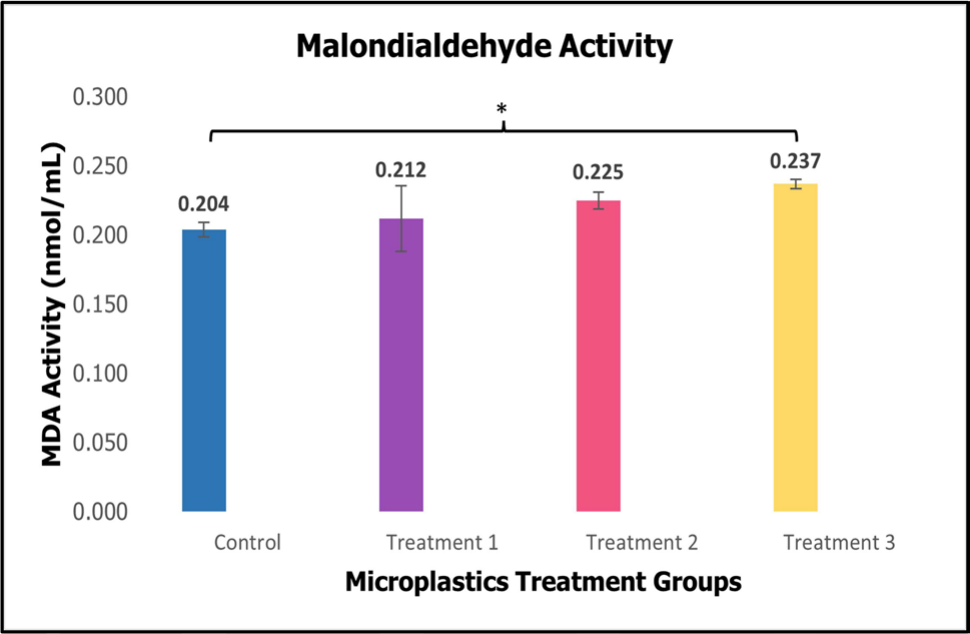
Activity of oxidative enzyme malondialdehyde (MDA) of sea cucumber *Holothuria scabra* after 60 days of microplastics treatments (mean ± SE). *Significant difference (*p* < 0.05, *p* = 0.037)

Malondialdehyde (MDA) exhibited a slight increase between the control and treatment groups, with a significant difference observed in Fig. 7.

### Histological analysis

**Fig. 8:**
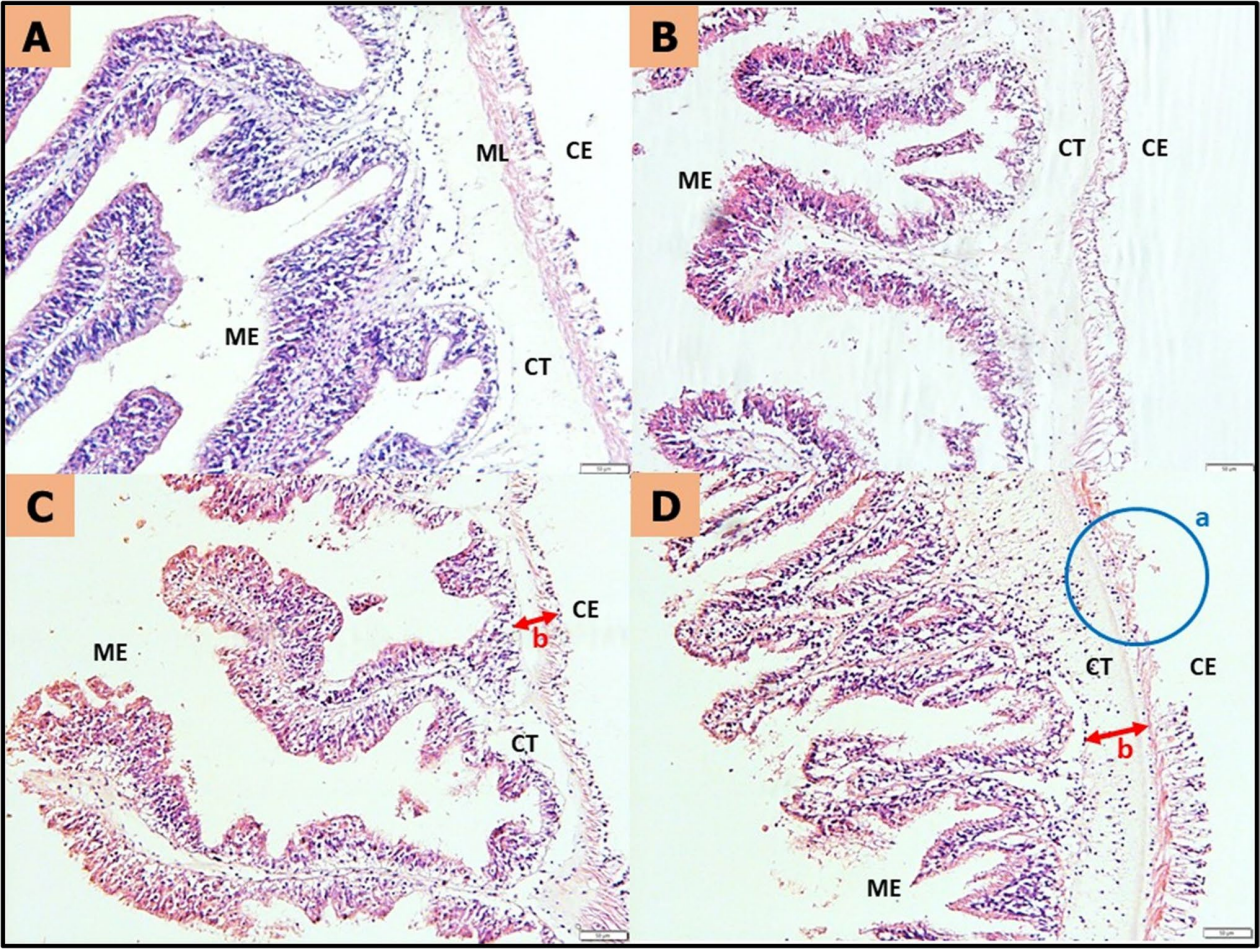
Histological sections in the gastrointestinal tract of *Holothuria scabra* after 60 days of MPs treatments. (A) Control (B) Treatment 1 (0.0003 g) (C) Treatment 2 (0.0005 g) (D) Treatment 3 (0.0042 g). CE – Coelomic Epidermis, ML – Muscular Layer, CT – Connective Tissues, ME – Mucosal Epithelium. [Magnification—10 ×, Scale Bar — 50 µm]

Histological examination revealed histopathological changes in the sea cucumber *H. scabra* inhabiting PMMA-MPs-contaminated environments. Figure 8 shows the damage sustained by *H. scabra* intestinal tract following 60 days of exposure to PMMA-MPs. The gastrointestinal tract of *H. scabra* was characterized by a coelomic epithelial lining, a longitudinal muscular layer, loose connective tissues, and well-arranged pseudostratified mucosal epithelium, as observed in Fig. 8A. In Fig. 8B, the intestinal structure showed no noticeable difference in Treatment 1. Exposure to higher concentrations of MPs in Treatment 2 and Treatment 3 resulted in diminished connective tissue in the intestines (Fig. 8C and D). Furthermore, the pseudostratified mucosal epithelium in Treatment 3 was damaged and disorganized, and the coelomic epithelial lining disintegrated, as observed in Fig. 8D.

**Fig. 9:**
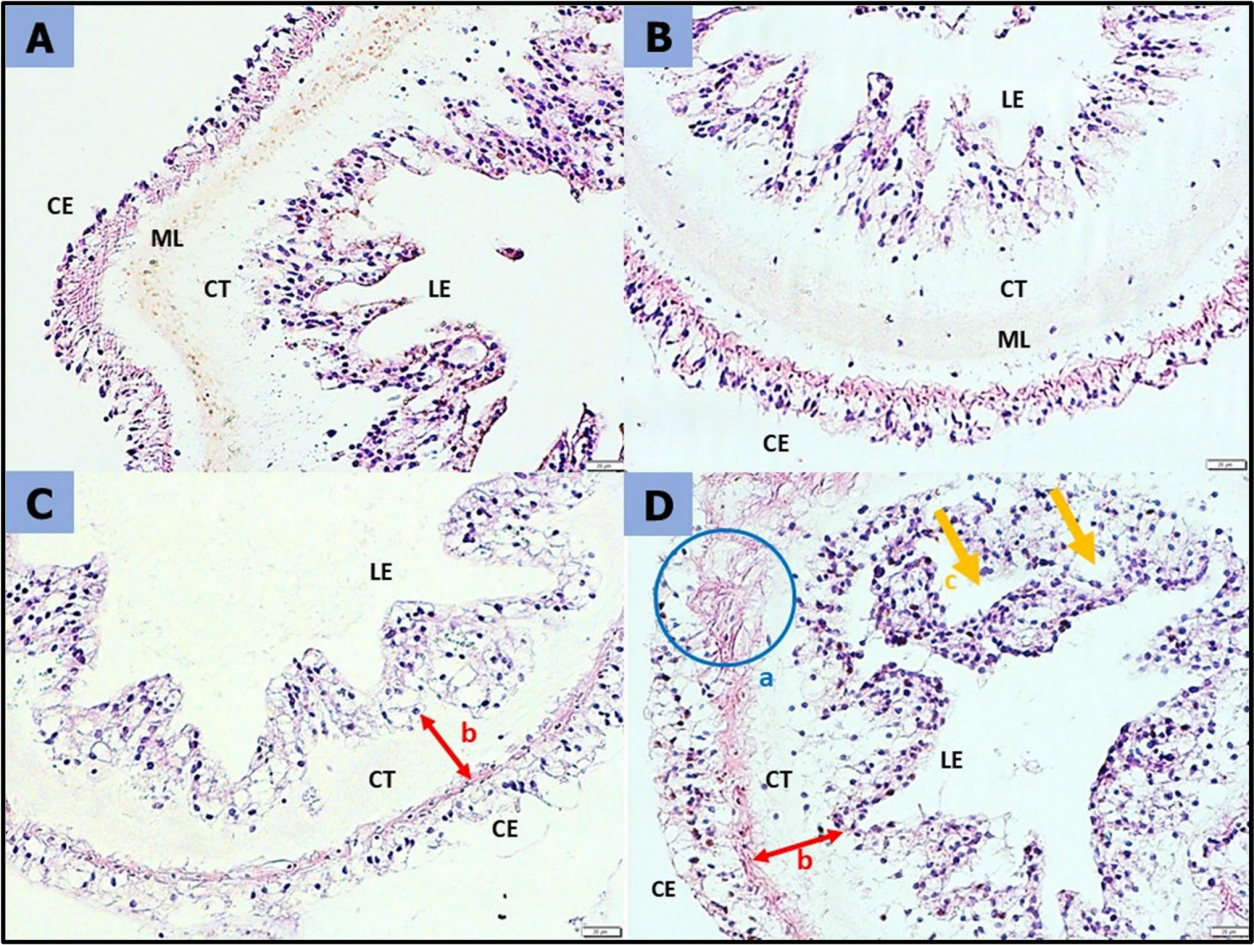
Histological sections in the respiratory tree of *Holothuria scabra* after 60 days of MPs treatments (× 20). (A) Control, (B) Treatment 1 (0.0003 g), (C) Treatment 2 (0.0005 g), (D) Treatment 3 (0.0042 g), CE — Coelomic Epidermis, ML — Muscular Layer, CT — Connective Tissues, LE—Lining Epithelium. [Magnification—20x, Scale Bar—20 µm]

Figure 9 shows the detrimental impact of 60 days of exposure to PMMA-MPs sustained by *H. scabra* respiratory tree. Figure 9A illustrates the epithelium of the respiratory tree cavity in H. scabra, comprising coelomic epithelium, muscular layer, connective tissue, and lining epithelium. The collagen fibers in the connective tissue were loose and well organized with a visible central cavity of the lining epithelium. In Treatment 1, there were no apparent histological changes, as depicted in Fig. 9B. However, with higher PMMA-MPs concentration, visible changes have been observed in Fig. 9C and D, particularly in the connective tissues and muscular layer, which becomes thinner and disintegrate. The lining epithelium was disorganized, the coelomic epithelial layer was damaged and vacuolation was apparent in Treatment 3 (Fig. 9D), illustrating the loss of cell components due to penetration of PMMA-MPs.

## Discussion

MPs are recognized as hazardous pollutants primarily due to their widespread presence and persistence in various habitats. The absorption of chemicals onto the plastic surface, resulting in a complex mixture of pollutants available to marine species, is facilitated by the high surface area to volume ratio of small particles and their non-polar surface (Sheela et al. 2021). The toxicity of MPs varies primarily depending on their size, as smaller particles can penetrate organisms more deeply. The density of MPs particles influences their position in the water column and their potential interaction with organisms, with denser or contaminated polymers posing a particular threat to benthic species (Desforges et al. 2015). Sea cucumbers typically feed on sediments enriched with organic matter, especially in regions where a high concentration of MPs particles builds up.

The specific growth rate (SGR) in sea cucumber *H. scabra* from three different PMMA-MPs treatment for 60 days of rearing period is presented in Table 1. In Fig. 1, the mean final weight of juveniles in control was higher (32.13 g ± 3.20) compared to treatment groups after 60 days (*p* < 0.05, *p* = 0.01). The weight gain percentage in the control group was highest (16.76% ± 0.35) as shown in Fig. 2. The specific growth rate of *H. scabra* differed significantly (*p* < 0.05, *p* = 0.03) among treatments as shown in Fig. 3. The highest SGR value was obtained from the control group at 1.22 ± 0.35%, while the lowest value was found in Treatment 3 at 0.47 ± 0.13% as depicted in Table 1. The findings demonstrated a significant impact of PMMA-MPs concentration on the sea cucumber’s final weight, specific growth rate, and survival rate. This is consistent with research by Liu et al. (2022), which showed that polystyrene nanoplastics and MPs adversely impact the final dry weight and weight gain of sea cucumber *Apostichopus japonicus*. However, Mohsen et al. (2021b) reported that microfibres did not significantly affect the survival rate and growth of sea cucumber *A. japonicus*, contradicting our current results. Previous studies have reported adverse effects of MPs ingestion on the growth of various other marine animals. The weight gain and body length growth of carp *Cyprinus carpio* larvae were suppressed by MPs made of polyvinyl chloride (PVC) (Xia et al. 2020). According to Bringer et al. (2021), seven days of exposure to MPs resulted in delayed growth retardation of oyster pediveliger larvae *Crassostrea gigas* for up to 28 days. In the control group with no PMMA-MPs treatment, sea cucumbers experienced optimal growth conditions with minimal stress since there were no inhibitory effects on growth. The occurrence of biofilm development in the tanks of the control and treatment groups also suggested the availability of extra food and nutrients such as microalgal and microbial cells, supporting the growth of juvenile sea cucumbers by facilitating easy access to nutrition in the enriched substrate (Rodrigues et al. 2023). In Treatment 2 and Treatment 3, sea cucumbers are likely to experience more adverse effects because higher concentrations of MPs may lead to physiological stress and metabolic disturbance significantly impacting their growth. While smaller MPs have a higher likelihood of entering the animal’s tissue, the larger MPs that are found in the animal feeds may result in lower ingestion rate values and energy digestibility, ultimately impacting growth performance (Shi et al. 2015).

The survival rate of *H. scabra* was notably influenced by the concentration of PMMA-MPs in the experimental treatments. The overall survival of *H. scabra* was 100% in control and Treatment 1, whereas it decreased to 75% in Treatment 2 and Treatment 3, experiencing the loss of 3 animals, respectively. The loss in Treatment 2 and Treatment 3 was linked to skin ulceration and lesion disease and subsequent evisceration, a physiological response in sea cucumbers triggered by stress or environmental disturbance. This is likely linked to MPs serving as vectors for Vibrio bacteria. *Vibrio splendidus* in particular, has been identified as a significant pathogen causing skin ulceration syndrome which heavily infect the sea cucumber, leading to mortality (Song et al. 2018; Wang et al. 2021). MPs in marine environment provide surfaces for microbial colonization. These bacteria can adhere to and form biofilms on MPs surface that facilitate their persistence and transport in the environment (Bowley et al. 2021). Organic materials such as leftover feed and fecal matter accumulate in the sediments creating nutrient-rich conditions that promote the proliferation of *Vibrio* species. Therefore, the ingestion of MPs with attached pathogens, combined with poor sediment quality, contributing to the infections and health deterioration in the affected sea cucumbers.

White spots developed on the body wall, rapidly affecting the entire integument, ultimately resulting in evisceration and fatalities. This could be due to the contaminated tanks whereby Treatment 2 and Treatment 3 had higher PMMA-MPs concentrations. The presence of additional PMMA-MPs in the sediment led to environmental pollution, leading to hypoxia stress in sea cucumbers themselves. The stress may weakened their health and potentially contributing to the development of this disease. This environmental factor caused the outbreak of cutaneous disease within the group since sea cucumbers are highly susceptible to sudden changes in external conditions. Evisceration, a defense mechanism commonly exhibited by sea cucumbers, involves the expulsion of internal organs as a reaction to extreme stress. In this study, the observed evisceration had increased vulnerability to mortality.

According to Huang et al. (2020), the accumulation of MPs can disrupt the homeostasis of animals as they persist in the body over an extended period, thereby elevating energy consumption and leading to malnutrition, which can potentially cause severe damage to organisms.

Sea cucumbers depend on their cellular and humoral innate immune responses when they are under hypoxic stress and are attacked by pathogens (Xue et al. 2015). These responses play a vital role in identifying and expelling invading microbes, as well as in repairing tissue. Acid phosphatase (ACP) activities were used as indicators for evaluating the immune status of *H. scabra*. ACP, as an intracellular lysosomal enzyme, involved in the immunological response, where it is responsible to eliminate and digest microorganisms and foreign substances (Yan et al. 2014).

As the concentration of PMMA-MPs in the ingested feed of juveniles increased, Fig. 4 depicted a rise in ACP activity with a significant difference. This elevation suggested a concentration-dependent response. The observed disruption in ACP was likely attributed to the increasing number of PMMA-MPs in the ingested sediment, which in turn triggered immune responses. This observation aligns with previous research, as noted by Mohsen et al. (2021b), where significant fluctuations in ACP activity were observed in the juvenile and adult sea cucumber *A. japonicus*, with a decreased in adults and an increased in juveniles. The developmental stage of sea cucumber appears to influence the activities of enzymes. Research involving other aquatic species also support the relationship between MPs exposure and ACP activity. For instance, exposure to 50 or 500 μg L^−1^ MPs containing cadmium showed considerably increased ACP activity in discus fish *Symphysodon aequifasciatus* (Wen et al. 2018). In a study particularly with pearl oyster *Pinctada fucata martensii*, the ACP activity of 1.5 mg/L and 15 mg/L polyvinyl chloride (PVC) MPs treatment groups were also increased (Lu et al. 2024). Additionally, the enzyme activity of ACP in brine shrimp *Artemia franciscana* increased with the increasing of MPs concentration after 14 days exposure period (Han et al. 2021). The responsiveness of ACP to environmental stimuli in sea cucumbers is evident, including hypoxic stress caused by oxygen deficiency and dietary supplementation of biofloc and fulvic acid (Chen et al. 2018; Dou and Wu 2023; Huo et al. 2018).

The ability of marine species to utilize ingested nutrients from food relies on the activities of digestive enzymes in their intestinal tract such as lipase. The activity of digestive enzymes not only signifies the digestive process, but also function as a biological indicator of the growth and health of the animal (Li et al. 2014). In comparison to the control with treatment groups, Fig. 5 showed an increased in lipase activity with a significant difference in the digestive tract of sea cucumber as the concentration of PMMA-MPs increased. This finding was consistent with a study on *A. stichopus*, where no significant changes were observed at different concentrations of polyethylene terephthalate (PET) MPs (particle size: 0.5–45 µm, 2–200 µm and 20–300 µm) (Zhang et al. 2023). As highlighted by Khan et al. (2017), the enzyme can degrade plastics polymer polyurethane (PU), suggesting that the lipase present in sea cucumber may play a role in lipid metabolism associated with MPs. This enzymatic activity could be linked to a stress response and detoxification mechanism to adapt to changes in environmental conditions. In contrast, polystyrene (PS) microspheres, a type of MPs had a substantial impact on lipase level of thick shell mussels *Mytilus coruscus* throughout the experiment (Wang et al. 2020). According to Susanto Barus et al. (2023), the activity of lipase in hard clam *Paphia undulata* was also significantly affected by PS-MPs treatments, either alone or in combination with heavy metals like copper and lead. Other than that, environmental conditions such as water temperature do influence lipase level in *A. japonicus*, with increases reaching its peaks at 20 °C (Sun et al. 2018).

MPs are recognized as hazardous due to their potential to generate free radicals, which can damage cellular macromolecules and disrupt physiological and biochemical processes (Alimba and Faggio 2019). This damage is closely associated with oxidative stress, a condition arising from an imbalance between oxidants and antioxidants in favor of oxidants. This imbalance occurs when the production of reactive oxygen species (ROS) overwhelms the antioxidant defense mechanisms. The excessive formation of ROS can lead to cellular damages.

Superoxide dismutase (SOD) served as the primary defense in the antioxidant system, catalyzed superoxide anion free radicals produced by activated coelomocytes to hydrogen peroxide (H_2_O_2_) or oxygen (O_2_) to protect cells from injuries. In the current experiment, the observed increase yet the difference did not reach statistical significance in SOD level in treatment groups indicated heightened oxidative stress, likely to be linked to a rapid rebound in oxygen uptake and consumption, along with an accelerated rate of reactive oxygen species (ROS) regeneration as shown in Fig. 6. Gu et al. (2023) noted that SOD activity in *A. stichopus* exhibited significant elevation in the initial exposure of PS-nanoplastics, however, it decreases as exposure time is prolonged suggesting the exhaustion of antioxidant defence. Various aquatic animals have been reported to experience disturbance in antioxidant enzymes due to MPs. Exposure to PS-MPs induces an increase in SOD activity in mussels, which reflects an adaptive response to mitigate oxidative stress and protect tissue from damage (Paul-Pont et al. 2016). In a study conducted by Ding et al. (2018), increased activity of anoxidative enzyme SOD in the liver of freshwater fish red tilapia *Oreochromis niloticus* was observed during exposure to MPs. Additionally, earlier studies have also demonstrated that sea cucumbers *Holothuria scabra* and *Apostichopus japonicus* also change SOD levels in response to environmental stressors such as temperature changes and salinity fluctuations. Higher temperature was associated with increased SOD levels, as reported by Kamyab et al. (2017); Wang et al. (2008); Yunwei et al. (2007). The SOD level increased in 35% salinity treatment in the first three hours of treatment and 25% salinity treatment exhibited a similar response for the first 72 h (Wang et al. 2008). Antioxidant activities particularly SOD in *H. scabra* play a crucial role in maintaining the oxidation-antioxidation equilibrium of sea cucumbers, reflecting the immune condition of animals. This suggests prolonged exposure to PMMA-MPs can weaken the antioxidant defense of *H. scabra* and increase susceptibility to infections, further impacting survival and growth rates.

When organisms encountering environmental stress, the balance of ROS was broken, resulting in lipid peroxidation. Malondialdehyde (MDA) is a toxic byproducts of lipid peroxidation, specifically the breakdown of polyunsaturated fatty acids by ROS. It serves as a dependable indicator of oxidative stress, known to cause damage to the cell membrane (Ayala et al. 2014). MDA is widely acknowledged as a biomarker for oxidative stress across many health disorders. Increased in the activity of the tested antioxidant enzyme MDA were observed. Although the effects were statistically significant, there was a trend for higher MDA activities in treatment 3, compared to control and other treatment groups, as shown in Fig. 7. This suggests that an increased oxidative stress brought on by PMMA-MPs exposure consequently promotes inflammation and cellular damage, potentially resulting in the breakdown of lipids. An excess of MDA level can occur when the antioxidant system is overworked, there may be an insufficient capacity to prevent lipid peroxidation. Similar response has been observed after MPs ingestion in previous studies. Mohsen et al. (2021b) reported that *A. japonicus* showed disruption of MDA levels in both juvenile and adult after 60 days of exposure. Lu et al. (2024) have also found that MDA content in pearl oyster *P. fucata martensii* was significantly higher in 15 mg/L dose PVC-MPs group compared to control group. High level of MDA in freshwater fish is said to induce oxidative stress due to the presence of MPs (Atamanalp et al. 2022).

Histological examination revealed histopathological changes in the sea cucumber *H. scabra* inhabiting MPs-contaminated environments. The elevated levels of SOD and MDA observed in this study provide supporting evidence for the histological changes of the intestines and respiratory tree, suggesting that oxidative stress induced by MPs was taking place in these tissues. Figure 8 and Fig. 9 show the damage sustained by *H. scabra*’s gastrointestinal tract and respiratory tree following 60 days of exposure to PMMA-MPs.

Sea cucumbers’ capacity to digest and absorp the nutrients from their feed relies significantly on the effectiveness of their gastrointestinal tract. The gastrointestinal tract of *H. scabra* exhibited distinct features, including coelomic epithelial lining, a longitudinal muscular layer, loose connective tissues, and well-arranged pseudostratified mucosal epithelium, as depicted in Fig. 8A. Following exposure to PMMA-MPs, histopathological examination uncovered changes in an inflammatory response and impaired integrity of the epithelial barrier in the intestine of sea cucumber. Disintegration of coelomic epithelial lining, reduced connective tissues, and disorganized arrangement of pseudostratified mucosal epithelium was observed in Fig. 8. Irregularly shaped MPs can cause physical damage to the intestinal tract due to sharp edges (Cole et al. 2019). Thus, this confirms that PMMA-MPs caused intestinal damage and inflammation, and damaged the intestinal folds. Impaired intestinal structure, particularly the mucus layer, would likely to affect immune function and raise the risk of intestinal inflammation since the intestinal epithelium serves as a crucial barrier between the immune system and external environment (Zhao et al. 2019). The degradation observed in intestine tissue suggests weak functions of digestion and absorptive functions under environmental stress, potentially impacting growth. Furthermore, intestinal tract is notably sensitive to stressors, leading to a various alterations including changes in normal protective microflora and reduced integrity of the intestinal epithelium (Zhao et al. 2019). Studies have demonstrated that exposure to MPs can induce damage to the tissue morphology of aquatic animals. The main intestinal damage in zebrafish *Danio rerio* included inflammation, bowel wall thinning, epithelial damage cracking of villi and splitting of enterocytes after exposure to MPs (Lei et al. 2018; Qiao et al. 2019). According to Zheng et al. (2022), the observed shedding of microvilli, increased in vacuolization and goblet cells, cytoplasmic damage dispersion and the loosening of connective tissue collectively signify that the exposure to PS-MPs has induced notable histopathological alterations in the intestines of the octopus *Amphioctopus fangsio*.

The respiratory tree in *H. scabra* serve as its unique respiratory organ, facilitating gas exchange between water and coelomic fluid (Gao and Yang 2015). Additionally, they play a role in osmotic regulation and provide space for the excretion of metabolized byproducts. PMMA-MPs have the potential to enter the coelomic fluid of sea cucumbers through the respiratory tree, where it could accumulate in the coelomic fluid with no way out since the haemal system of sea cucumbers is a closed system (Mohsen et al. 2020). The respiratory tree was composed of coelomic epithelium, muscular layer, connective tissue, and lining epithelium as shown in Fig. 9A. The collagen fibers in the connective tissue were loose and well organized with a visible central cavity of the lining epithelium. In our current study, a clear indication of loss of cell components, vacuolation, and damaged cell layer was apparent in treatment 2 (Fig. 9C) and treatment 3 (Fig. 9D) after 60 days of treatments. According to Mohsen et al. (2021a), abnormal changes in respiratory tree tissues including erosion of epithelium, vacuolation, and injuries in all tissue layers of *A. japonicus* were observed due to MPs exposure. Damages of the respiratory trees were also induced by environmental stressors such as mercury and salinities variation (Geng et al. 2016; Telahigue et al. 2020).

PMMA MPs ingestion highlights the implications of MPs in sea cucumber *H. scabra*, as demonstrated by the complex interactions between growth performance, immune responses, oxidative stress, and histopathological changes. These findings will help to further assess the toxicity studies of MPs and emphasized the need for effective mitigation strategies to address the current issues of MPs pollution in the marine environments as well as to improve the health of marine species and ecosystem. However, understanding environmental factors which may influence MPs toxicity, is crucial in developing effective conservation and management strategies to mitigate the impacts of MPs pollution in marine environment. Further work is required to enhance better understanding the risks associated to various types MPs polymers and their interaction with other environmental stressors. It is important to improve general wellbeing and productivity of sea cucumber population as well as the marine ecosystem, which are currently facing the impacts of MPs pollution.

## Conclusion and recommendation

In conclusion, the exposure of sea cucumber *H. scabra* to PMMA-MPs has significant implications for its growth, survival, and overall health. The study revealed that different concentrations of PMMA-MPs led to variations in the specific growth rate and survival rate, exhibited significant differences (*p* < 0.05) with the control group displaying the highest SGR value of 1.22 ± 0.35%, and Treatment 3 has the lowest SGR value (0.47 ± 0.13). This also emphasizes the potential risks posed by MPs, including environmental stress, skin ulceration, and lesions, which may result in evisceration and fatalities. Mortality was observed in Treatment 2 and 3, respectively. Additionally, the study highlighted the impact of MPs on immune responses and oxidative stress which underscore the damage caused at the cellular level. A disruption in enzyme assays was observed (*p* < 0.05) in acid phosphatase, lipase, superoxide dismutase, and malondialdehyde. Higher concentrations of PMMA-MPs caused histological changes in the intestines and respiratory trees of sea cucumber *H. scabra*. This study signifies the first exploration into the toxicity of MPs specifically targeted at *H. scabra*.

## Data Availability

Data will be made available on request.
